# Women’s participation in breast cancer screening in France – an ethical approach

**DOI:** 10.1186/1472-6939-15-64

**Published:** 2014-08-16

**Authors:** Grégoire Moutel, Nathalie Duchange, Sylviane Darquy, Sandrine de Montgolfier, Frédérique Papin-Lefebvre, Odile Jullian, Jérôme Viguier, Hélène Sancho-Garnier

**Affiliations:** 1 Assistance Publique Hôpitaux de Paris, Hôpital Universitaire Georges Pompidou Corentin-Celton, Université Paris Descartes, 92130 Issy-les-Moulineaux, France; 2 PRES Sorbonne Paris Cité/Ecole des hautes études en santé publique EHESP, Equipe MOS, Management des organisations de santé, 75014 Paris, France; 3 Université Paris Descartes, EA 4569, Faculté de médecine, 75006 Paris, France; 4 Institut de Recherche Interdisciplinaire sur les enjeux Sociaux (IRIS) Cnrs/Inserm/EHESS, Université Paris-Est Créteil, 93017 Bobigny cedex, France; 5 Institut médico-légal, Centre hospitalo-universitaire de Caen, 14033 Caen cedex, France; 6 Inserm, U1086 Cancers et préventions, Faculté de Médecine, 14076 Caen, France; 7 Institut National du Cancer, 92513 Boulogne-Billancourt cedex, France; 8 Université de Montpellier, Laboratoire Epsilon, 34000 Montpellier, France; 9 Unité de Médecine sociale, Hôpital Universitaire Georges Pompidou Corentin-Celton, AP-HP, 4 Parvis Corentin Celton, 92130 Issy-les-Moulineaux, France

**Keywords:** Breast cancer screening, Organized programme, Ethics, Information

## Abstract

**Background:**

Breast cancer is a major public health challenge. Organized mammography screening (OS) is considered one way to reduce breast cancer mortality. EU recommendations prone mass deployment of OS, and back in 2004, France introduced a national OS programme for women aged 50–74 years. However, in 2012, participation rate was still just 52.7%, well short of the targeted 70% objective. In an effort to re-address the (in) efficiency of the programme, the French National Cancer Institute has drafted an expert-group review of the ethical issues surrounding breast cancer mammography screening.

**Discussion:**

Prompted by emerging debate over the efficiency of the screening scheme and its allied public information provision, we keynote the experts’ report based on analysis of epidemiological data and participation rate from the public health authorities. The low coverage of the OS scheme may be partly explained by the fact that a significant number of women undergo mammography outside OS and thus outside OS criteria. These findings call for further thinking on (i) the ethical principles of beneficence and non-malfeasance underpinning this public health initiative, (ii) the reasons behind women’s and professionals’ behavior, and (iii) the need to analyze how information provision to women and the doctor-patient relationship need to evolve in response to scientific controversy over the risks and benefits of conducting mammographic screening.

**Summary:**

This work calls for a reappraisal of the provision of screening programme information. We advocate a move to integrate the points sparking debate over the efficiency of the screening scheme to guarantee full transparency. The perspective is to strengthen the respect for autonomy allowing women to make an informed choice in their decision on whether or not to participate.

## Background

### A need for an expert group review on the ethics of the screening programme

In population-based screening, individual benefits are random and few, as the desired outcome hinges on collective benefits. Breast cancer screening is offered to asymptomatic women who may then have to contend with a sudden change from ‘well’ to ‘ill’, with all the ensuing psychological, psychosocial and economic repercussions. This outcome can lead to unnecessary examinations, sometimes with secondary effects in subjects wrongly diagnosed as “positive”. That said, the success and relevance of any screening programme hinges on acceptance of a procedure and compliance with its criteria [1]. Democratic societies are founded on respect for autonomy, especially in the medical domain. Today, compulsory and imposed public health measures may no longer be accepted without total transparency and understanding.

In its role as the body responsible for deploying the breast cancer screening policy programme since 2004, the National Cancer Institute (INCa), a French government agency, led an expert-group review of the ethical issues surrounding this public health process. Prompted by emerging debate over the efficiency of the screening scheme and the allied public information provision, this work resulted in a report published in late-2012 [2]. The group’s thinking, reported here, was based on analysis of epidemiological data and OS participation rate from the French public health authorities. The aim was to address the ethics issues surrounding international debate over programme benefits and risks in order to refocus the information given to women onto greater respect for autonomy in their decision on whether or not to participate.

### Implementation of the French breast cancer screening programme

**Figure 1:**
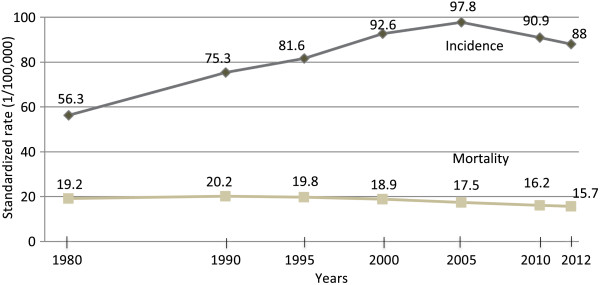
**Evolution of mortality and incidence rates (1/100,000) in France from 1980 to 2012**[3,4]**.**

Breast cancer is a major public health challenge. It is the most common cancer among women in France, accounting for an estimated 48,763 new cases and 11,886 deaths in 2012 [3]. According to French National Institute of Statistics and Economic Studies (INSEE), breast cancer incidence increased 1.4% per year over the 1980–2012 period (Figure 1) [3,4].

Organized mammography screening (OS) is considered one way to reduce breast cancer mortality [5]. EU recommendations advocate broad deployment of OS [6]. France introduced OS throughout the country in 2004 under the First and Second French Cancer Plans (2003–2007 and 2009–2013). Based on recommendations in response to the risk factors (gender and age), the programme invites women aged 50–74 years to attend screening (breast examination and a mammography) every two years. The scheme was established according to specifications and good practices, including double reading of each negative mammogram and, when necessary, an ultrasound examination. The screening programme is organized at regional level by management centres that send out individual postal invitations inviting women to participate by attending accredited radiological centres. The invitation letter does not specify any pre-defined appointment date ― women are free to make an appointment in a list of accredited centres enclosed with the letter of invitation. The principle of informed decision-making is thus respected. Non-responder or non-participating women get a follow-up letter within the next six months. Results are handled and monitored by the management centres. The process targets a population of nearly 9 million women, excluding those presenting a high risk of breast cancer due to family history, genetic predisposition, personal history of thoracic irradiation or at-risk of benign tumours.

## Discussion

### Questions arising from the programme participation rate

**Figure 2:**
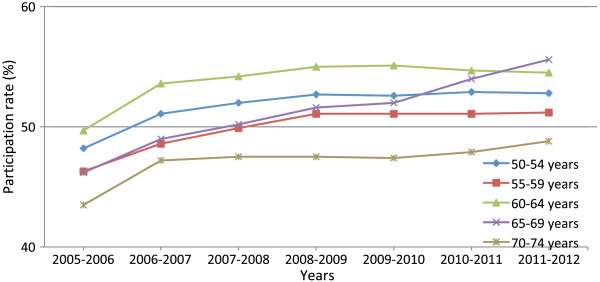
**OS participation (%) by age bracket**[8]**.**

Participation rate is one indicator for assessing the performance of a screening programme. Based on epidemiological data and experts’ consensus recommendations, European guidelines set a target OS participation level of 70% as acceptable whereas 75% is the desirable level [7]. The 70% target level was set on the basis that a high rate of participation among invited women was necessary in order to maximize the mortality benefits of population-based breast cancer screening in a cost-effective manner. However, according to French Institute for Public Health Surveillance (InVS) data, participation in the French OS programme was only 52.7% in 2012, with no significant increase recorded since 2007 (Figure 2) [8]. The data also highlight strong regional disparities, with rates ranging from 67.6% in the Loire-Atlantique region down to 27.6% in Paris. Fifty-four French *départements* report over 55% participation, while 21—including six of the eight *départements* of the Greater Paris region—report less than 50% participation.

**Figure 3:**
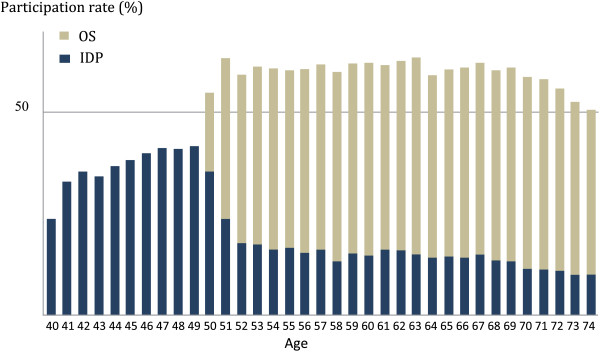
**Mammography participation (%) by age bracket under organized breast cancer screening (OS) and individual detection procedures from 2008 to 2009**[9]**.**

However, a significant number of mammographies that should be performed in OS are still prescribed outside the programme, and thus outside the OS inclusion criteria (Figure 3) [9]. Mammographies performed outside OS and its criteria should not normally involve women other than those presenting high risk factors (family history, genetic predisposition, personal history of thoracic irradiation or at-risk benign tumours) or those with clinical symptoms. Among the women aged 50–74 who undergo mammographies outside OS, only 7–8% would be diagnosed or monitored as belonging to the high-risk population, which leaves more than 90% that would otherwise meet the OS criteria. As these extra-OS procedures are performed outside accredited management centres, the data does not enter into the epidemiological evaluation of OS, which thus further lowers the OS participation rate. Under the French health insurance system, mammographies are always reimbursed, which may explain why a number of women meeting the criteria do not opt into the OS programme.

The 2011 report from the French National Authority for Health (HAS) [9] singled out limitations to maintaining this situation in terms of public health and medical effectiveness. Indeed, it was shown that a full transition to OS was preferable in terms of both number of cancers detected and costs involved. Further, the OS programme corresponds to standardized procedures, including traceability for epidemiological analysis, quality of radiological apparatus, and double reading of radiographies. Mammographies performed outside OS do not offer the same level of guarantees in a process that is not evaluable and thus less legitimate. The same report [9] also showed that in the absence of double reading of radiographies, 1.4% of abnormal images were missed, there was a higher rate of false-negatives, and OS screening structures were underused which reduces the efficiency of the programme. Furthermore, mammographies performed outside OS incur additional collective costs, as all health expenditures in France are reimbursed by the healthcare system. This is a situation specific to France, which has opted not to apply pressure to participate in OS and not to financially penalize women who opt to perform mammography outside the OS. This, again, is part of a policy designed to respect the principle of autonomy, and also part of a continuum, as many women have already had a mammography without medical indications before reaching their 50s, and the community reimburses them.

The issue is rooted in a medical-economic dimension, at a time when the solidarity-based national insurance system supporting these non-OS practices is facing increasing pressure.

Our group emphasized the need to raise women’s awareness of the importance of the criteria established in the OS programme, both in terms of age and intervals between examinations and in terms of the quality of the procedures for accreditation, all of which is linked to benefit/risk ratio. It is clearly important that communication to candidate participants should stress these elements.

### Barriers to participation in organized screening

Studies in the French context [10,11] have identified a number of barriers to participation, some of which explain why a number of women do not undergo mammographies:

• The feeling of not being concerned, due to an absence of symptoms and feeling healthy;

• The sense of fatalism over cancer and the belief that it is impossible to prevent its outcome;

• Lack of time for personal healthcare (for social, family or medical reasons);

• Other life constraints or priorities (day-to-day, housing problems, etc.);

• Absence of regular follow-up by a medical gynaecologist or GP;

• Women foregoing basic healthcare for financial reasons are less likely to participate in screening;

• Language barrier and lack of knowledge of the healthcare system.

• A study by Duport [11] in 2008 ruled out low geographical accessibility to a radiologist as a problem due to the existence of neighbourhood health structures in France.

Other barriers explain why a number of women do not switch to OS:

• Lack of a clear distinction between diagnostic and screening mammographies;

• Perception of OS as a highly impersonal procedure managed by an administrative apparatus with poorly-identified structures, and the negative view some people have of any publicly-run process;

• Organizational issues (especially lack of flexibility when making appointments, etc.).

• The feeling of not being concerned, due to perceptions that the programme is a social provision targeting financially disadvantaged people;

• The preference for a personal relationship with a physician rather than being invited by mail by the management centres in charge of the OS programme.

Our group underlined the importance of targeting the information according to these two categories of barriers. For the first category, the accent should be placed on educating the target audience on cancer, screening and access to the healthcare system. For the second category, the focus should be on the specificities of the OS programme and on the importance of respecting the OS-framework criteria.

This conclusion challenges both the content of the tools used in the information campaigns and the place given to physicians (GPs and/or gynaecologists) in the OS process, which as a group had not been sufficiently involved in organizing the OS programme at its outset.

### Controversies over the benefit-risk ratio

Debate over the level of efficacy of screening continues to rage, and scientific controversy surrounding overdiagnosis and overtreatment raises further questions over the whole communication strand of the OS system. Historically, the efficacy of breast cancer screening in terms of reducing mortality was established based on the results of randomized trials [5], and was only later brought into question [12,13]. These trials aimed to investigate not just the impact of screening on breast cancer mortality but also the evolution of risks inherent to screening, e.g. false-negatives as well as false-positives. More than 10 randomized trials carried out in various countries since 1963 showed that OS led to a relative reduction in breast cancer mortality of 15–32% after 7–10 years of follow-up in the 50–69 age group enrolled in these trials. However, in the early 2000s, fresh analysis sparked major controversy by showing methodological weaknesses in some of these trials and challenging the extent of the benefit claimed in terms of reduced breast cancer mortality [14,15]. A re-analysis of results by the US Preventive Task Force in 2009 [16] based on nine trials showed a 14% reduction in mortality for the 50–59 age group and a 32% reduction for the 60–69 age group. Other studies in the last 20 years have estimated how far screening has helped reduce breast cancer mortality in their populations [17-22], but with results that put the reduction directly attributable to screening at 3–20% depending on the country. According to a 2012 UK study [23], the number of women who need to be screened every 2 years for 10 years to avoid one death is 1610 for women aged 45–55 and around 750 for women over 60. Since then, Marmot et al. [24] in 2013 published the full report on the benefits and harms of screening as commented on by leaders in the field in the same issue of the journal. All these studies, and the reported uncertainties, serve to fuel the controversy surrounding the efficacy of screening.

The benefits of breast cancer screening have to be balanced against its harms, particularly overdiagnosis—which is defined as cases where screening detects a cancer that would not have become clinically apparent in the woman’s lifetime had she not been screened—and false-positive results—which occur when mammographic images are wrongly diagnosed as positive (possible malignancy). In both cases, further examinations then become necessary that are liable to cause adverse effects and distress to patients. This can lead to unnecessary treatments and even mutilation (mastectomy), and thus a reduction in quality of life. Estimated overdiagnosis rates vary greatly from one country to another. A study using the Isère *département* cancer registry in France estimated that overdiagnosis in cancer detected by mammography screening only was 3.3% for invasive cancers and 31.9% for *in situ* carcinomas [25]. A review from an independent UK panel on breast cancer screening stressed that for every breast cancer death prevented, about three overdiagnosed cases will be identified and treated [23]. Puliti et al. [26] reported that estimations contain a number of “biases” and variability criteria and concluded that overdiagnosis in mammographic screening for breast cancer in Europe is somewhere in the range 1% to 10%. However, overdiagnosis rate was estimated at 29% in the Cochrane review [27] and at 52% in a systematic review of countries with OS programmes [28]. For false-positives, a recent literature review showed that the estimated risk in women aged 50–69 ranges from 8% to 21% in Europe [29].

Interval cancers, i.e. cancer diagnosed between successive mammographies, are also an important concern, whether a tumour is present but not detected (false negative) or whether a fast-growing tumour appears between screening rounds. In France, two-year interval cancer rate was estimated at 15.3 per 10,000 women screened in 2004 in the OS programme with two-view mammography [30]. The existence of interval cancers may influence both patients’ and health professionals’ perceptions and trust in the benefit of the screening programme, and may make it necessary to give specific information on the uncertainties involved. Participation does not necessarily imply knowledge and understanding of all the aspects involved in a screening procedure [31].

Finally, the potential damaging effects linked to irradiation and the existence of radio-induced cancer also have to be factored in. While the risk exists, it appears to be low [32]. Efforts to quantify radiation-induced cancers following participation in breast cancer mammography screening remain controversial and difficult to produce. Epidemiological studies on this topic have mainly consisted in predicting numbers of radiation-induced cancers. It was found that the risk of cancer associated with mammographic irradiation is higher when exposure occurs at younger ages but decreases with increasing age [33]. Finally, the EU directive on ‘health protection of individuals against the dangers of ionizing radiation through medical exposure’ states that medical exposure has to be justified and is therefore prohibited in the absence of any indication [34]—a recommendation that, in practice, is evidently not always followed.

On the issue of the balance between benefits and risks, our group underlined the importance of revising the information given to women so as to integrate the context of uncertainty, particularly the uncertainty tied to overdiagnosis and the existence of interval cancers.

### Between promotion and information on screening

Women have a fairly positive perception of breast cancer screening [11,35] thanks to the communication efforts pioneered by the media, health authorities and physicians. However, growing concern over possible harm from breast cancer screening have spurred calls to provide more balanced and unbiased information to ensure respect for autonomy and the principle of informed choice [36]. Optimizing informed choice for women thus requires an evolution in the information delivered both by professionals and *via* national campaigns.

It has been shown that successful implementation of OS is heavily reliant on physicians, who play a key role in counselling women [11,37]. It is thus vital for health professionals themselves to be well informed so that they can pass on valid up-to-date data. A recommendation from the French health authorities [HAS] in 2011 [9] stressed the need for health professionals to get refresher training on the content of the information to be delivered to women. This should optimize balanced information and help avoid a one-sided positive discourse on screening.

Our group of experts underlined that the information delivered to candidate women must include all potentially negative effects of screening. Any contrary approach would be unacceptable. Thus, in the 2013 campaign on information for breast cancer screening, the leaflet produced by the INCa included a new section describing the potential undesirable effects [38]. The brochure was designed by the communication department in association with patient organizations, with our group’s coordinator relaying their recommendations. The innovation lies in the paradox where those in charge of promoting OS are at the same time responsible for designing the information that may prompt women to elect not to participate. The brochure describes the main benefits of participation as the estimated 15–21% reduction in breast cancer mortality and the quality of the medical monitoring involved. The risks were introduced in the leaflet under a chapter headed “drawbacks”. First, this “drawbacks” section states that mammography can detect anomalies requiring additional tests whereas cancer is not necessarily diagnosed―a situation referred to as “false positives”. Second, it states the case for treating slow-evolution cancer that may have had little or no incidence on the woman’s life. It explains that as today’s science cannot dissociate between slow and rapid-evolution cancers (10 to 20% of all cancers detected); the policy adopted is to treat all cases, which creates potential overtreatment. Third, the brochure refers to the existence of a low risk linked to exposure to irradiation during the mammography exam. This risk is estimated at 1 to 5 deaths per 100,000 women. Thus, this is the first French OS promotion campaign to integrate downside elements of information essential for women to make an informed choice. This is an evolution designed to instil greater transparency. This trend is in line with the stance taken in other countries, such as the UK where the National Health Service [39] produced a website entitled ‘Informed choice about cancer screening’ whose content on the allied benefits and harms was co-constructed with a citizen “jury” [40]. The website gives access to the latest scientific data, offers a new approach to developing information on cancer screening, and proposes a leaflet including the limits of benefits balanced with the risks that is sent out with the invitation letter. The balance is also a trade-off between the risk of having cancer (with a better prognosis if detected early) and the downside effects linked to screening. In 2013, a study by Hersch et al. [35] showed that a number of women would accept a risk linked to screening (depending on the magnitude of the overdiagnosis percentage presented to them) and not change their commitment towards screening.

### Between collective interests and individual liberty

These elements strongly echo the biomedical ethics principles of beneficence and non-malfeasance established by Beauchamp and Childress [41]. The principle of beneficence, applied to screening, means that it must show sufficient benefits (i.e. early detection improves cancer prognosis) while also contributing to individual well-being and quality of life rather than only to a reduction in breast cancer mortality. The principle of non-malfeasance underlines the need for vigilance on harmful effects and the obligation to regularly evaluate and communicate potentially damaging effects through an analysis of practices, and to do everything possible to minimize them.

The context of uncertainty raises questions over the good practices and guidelines to be applied for informing women. It is important to make the distinction between promotion of screening and information on screening [2]. As the two notions are not mutually exclusive, care should, however, be taken to not neglect or distort information. Since the French law of 4 March 2002 [42], patient information has been enshrined as a right in a process where the individual must be able to make an informed choice. This right is in line with the ethical principle of autonomy, which requires that physicians and other healthcare professionals must allow patients to make their own decisions on healthcare choices, especially for preventive care decisions [43]. The concept of informed choice takes a different shape depending on whether the context is screening or medical care. In the therapeutic approach, the objective is individual benefit for the patient, and the associated risks are generally accepted when balanced against the risk of letting a disease worsen. In breast cancer screening, the situation is reversed, since to achieve a collective benefit, individuals have to accept potential risks without being certain of gaining individual benefit [44].

In any public health action, the benefit sought is primarily collective, sometimes at the risk of harm to individuals. Such actions have long been seen as necessarily ethical, since they are for the greater good of the population. However, the picture has been shaken up now that individual risks in public health programmes are less accepted due to a general attitude moving away from paternalism and toward a strengthening of decision-making autonomy and informed choice, which consequently entails greater transparency on the risks involved in order to deliver fair information [45]. In this context, are women irresponsible if they opt out of OS? In the current state of scientific knowledge, and as long as OS is seen to have benefits in terms of mortality, non-participation could be viewed as counter to the wider public interest. However, as the benefit to the population appears to be low and controversial (unlike, for example, immunization campaigns against measles and tuberculosis), it appears difficult to stigmatize non-participating women and invoke the concept of irresponsibility.

All this illustrates the existence of tension between the interests or protection of the community, respect for individual liberty, and citizen responsibility. There is a political issue centred on criteria guiding the decision whether or not to promote and maintain an organized screening programme. This question was recently addressed by the Swiss Medical Board [46,47] that stressed the need for a public health programme that does not highlight more benefits than harms so as to at least provide clear and unbiased information.

## Summary

Organized screening carries stakes tied to the convergence between users’ rights, public health issues, and notions of collective ethical responsibility. The efficiency of the screening scheme is under debate and thus requires transparency. The challenge is to provide comprehensive and intelligible information (including rationale, goals, notions of collective responsibility, state of scientific knowledge, and areas of uncertainty over the benefit-risk balance) enabling women to make an informed choice on whether to participate in the programme without adding confusion or hampering the public health objective of reducing breast cancer mortality.

## Abbreviations

GRED: Groupe de Réflexion sur l’Ethique du Dépistage; HAS: French National Authority for Health; INCa: French National Cancer Institute; INSEE: French National Institute of Statistics and Economic Studies; InVS: French Institute for Public Health Surveillance; OS: Organized mammography screening.

## Competing interests

The authors declare that they have no competing interests.

## Authors’ contributions

All GRED members participated in the identification and analysis of ethical issues surrounding breast cancer screening in the OS programme. GM, ND, SD, SdM, FPL, OJ, JV, HSG were involved in compiling the epidemiological/participation data borrowed from the InVS and reviewing the literature. GM, ND, SD wrote the paper. SdM, FPL, OJ, JV and HSG critically revised the manuscript. All authors approved the final version of the manuscript before submission.

## Pre-publication history

The pre-publication history for this paper can be accessed here:

http://www.biomedcentral.com/1472-6939/15/64/prepub
